# How the Position of Substitution Affects Intermolecular Bonding in Halogen Derivatives of Carboranes: Crystal Structures of 1,2,3- and 8,9,12-Triiodo- and 8,9,12-Tribromo *ortho*-Carboranes

**DOI:** 10.3390/molecules28020875

**Published:** 2023-01-15

**Authors:** Kyrill Yu. Suponitsky, Sergey A. Anufriev, Igor B. Sivaev

**Affiliations:** 1 A. N. Nesmeyanov Institute of Organoelement Compounds, Russian Academy of Sciences, 28 Vavilov Str., Moscow 119334, Russia; 2 Basic Department of Chemistry of Innovative Materials and Technologies, G.V. Plekhanov Russian University of Economics, 36 Stremyannyi Line, Moscow 117997, Russia

**Keywords:** *ortho*-carborane, iodo derivatives, X-ray structure, I⋯I dihalogen bond

## Abstract

The crystal structures of two isomeric triiodo derivatives of *ortho*-carborane containing substituents in the three most electron-withdrawing positions of the carborane cage, 1,2,3-I_3_-1,2-C_2_B_10_H_9_, and the three most electron-donating positions, 8,9,12-I_3_-1,2-C_2_B_10_H_9_, as well as the crystal structure of 8,9,12-Br_3_-1,2-C_2_B_10_H_9_, were determined by single-crystal X-ray diffraction. In the structure of 1,2,3-I_3_-1,2-C_2_B_10_H_9_, an iodine atom attached to the boron atom (position 3) donates its lone pairs simultaneously to the σ-holes of both iodine atoms attached to the carbon atoms (positions 1 and 2) with the I⋯I distance of 3.554(2) Å and the C-I⋯I and B-I⋯I angles of 169.2(2)° and 92.2(2)°, respectively. The structure is additionally stabilized by a few B-H⋯I-shortened contacts. In the structure of 8,9,12-I_3_-1,2-C_2_B_10_H_9_, the I⋯I contacts of type II are very weak (the I⋯I distance is 4.268(4) Å, the B8-I8⋯I12 and B12-I12⋯I8 angles are 130.2(3)° and 92.2(3)°) and can only be regarded as dihalogen bonds formally. In comparison with the latter, the structure of 8,9,12-Br_3_-1,2-C_2_B_10_H_9_ demonstrates both similarities and differences. No Br⋯Br contacts of type II are observed, while there are two Br⋯Br halogen bonds of type I.

## 1. Introduction

The ability of halogens to form complexes with various electron pair donors was discovered over two hundred years ago [1,2,3], and the Nobel Prize laureate Odd Hassel provided crystallographic proof for the existence of such a bond, interpreting it as a charge-transfer interaction more than fifty years ago [4,5]. However, only at the beginning of the 21st century has halogen bonding grown from a scientific curiosity to one of the most interesting and actively studied non-covalent interactions for the construction of supramolecular assemblies [6,7,8,9,10].

This progress has been largely due to a better understanding of the principles on which the strength of the halogen bond depends. The performance of the halogen bond largely depends on the degree of polarization of the halogen atom; that is, the greater the positive electrostatic potential of the σ-hole, the more efficient the halogen bond donor will be [10,11,12]. The value of the positive potential of the σ-hole depends on the ability of the halogen atom to be polarized, which decreases in the following order: I > Br > Cl >> F [13,14]. The value of the positive potential of the σ-hole can be enhanced due to the electron-withdrawing ability of the fragment to which the halogen atom is attached. 

For a halogen atom to be an electron acceptor in order to form a halogen bond, it must be bonded to an electron-withdrawing atom or group. Therefore, the *sp* hybridization of carbon atoms bearing a halogen is favored over *sp^2^* followed by *sp^3^* hybridization [15,16]. The hybridization of the carbon atom can be compensated by the electron-withdrawing effect of fluorine atoms, as evidenced by the close values of the σ-hole potential of the corresponding iodine atoms in 1-iodoethynyl-4-iodobenzene and 1,4-diiodoperfluoro-benzene (172 and 169 kJ/mol, respectively) [16]. The strength of the halogen bond is highly correlated with the degree of iodobenzene fluorination [17]. Therefore, it is not surprising that 1,4-diiodoperfluorobenzene and its analogs are widely used in the design of halogen-bonded supramolecular systems [18,19,20,21,22,23,24,25,26,27,28], although arylacetylene iodides also play an important role [16,29,30,31,32,33,34,35]. In the absence of other electron density donors, the iodine atoms in these compounds are also able to play this role, which leads to the formation of I⋯I dihalogen bonds [36,37,38,39], and the number of such bonds, as a rule, increases with the number of iodine atoms in the molecule [40].

**Figure 1:**
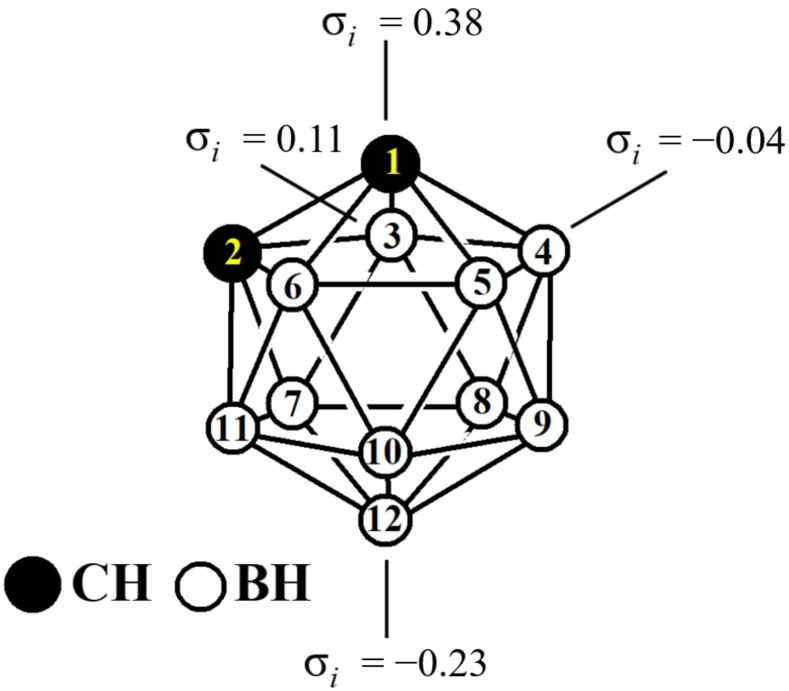
Atom numbering atoms and Hammett constants σ_i_ in *ortho*-carborane.

Icosahedral carboranes C_2_B_10_H_12_ are another class of compounds whose derivatives are promising as halogen bond donors. The predicted strength of the halogen bonds with the same electron donor (based on the σ-hole potential) is larger for *C*-vertex halogen-substituted carboranes than for their organic aromatic counterparts [41,42,43]. In contrast to the iodo aromatics, wherein all iodine atoms are equivalent, in the iodo derivatives of *ortho*-carborane iodine atoms, depending on their position, they can act preferentially as an acceptor or a donor of a halogen bond. A typical example is 1,12-diodo-*ortho*-carborane, in which one of the iodine atoms is bonded to the most electron-withdrawing position of the carborane cage (position 1), and the second to the most electron-donating position (position 12) (Figure 1) [44]. The first of them is an electron acceptor, and the last one is a donor, which form an ideal intermolecular I⋯I dihalogen bond of type II [45].

In this contribution, we studied intermolecular bonding in two isomers of triiodo-*ortho*-carborane containing substituents in the three most electron-withdrawing positions of the carborane cage (1,2,3) and the three most electron-donating positions (8,9,12); in addition, a comparative analysis of the crystal packings of the 8,9,12-triiodo and 8,9,12-tribromo derivatives of *ortho*-carborane was performed.

## 2. Results and Discussion

To date, a number of iodo derivatives of *ortho*-carborane have been synthesized, and the structures of a dozen of them have been established by single-crystal X-ray diffraction. The derivatives with a high degree of substitution such as 8,9,10,12-I_4_-1,2-C_2_B_10_H_8_ [46], 4,5,7,8,9,10,11,12-I_8_-1,2-C_2_B_10_H_4_ [47], and 3,4,5,6,7,8,9,10,11,12-I_10_-1,2-C_2_B_10_H_2_ [47], as in the case of iodo-aromatics, are characterized by the formation of numerous intermolecular I⋯I dihalogen bonds varying from 3.74 to 4.05 Å. In contrast to the polyiodo derivatives, no intermolecular dihalogen bonds were found in any of the isomeric monoiodo derivatives of *ortho*-carborane 1-I-1,2-C_2_B_10_H_11_ [45], 3-I-1,2-C_2_B_10_H_11_ [48], 8-I-1,2-C_2_B_10_H_11_ [49], and 9-I-1,2-C_2_B_10_H_11_ [47].

As for the diiodo derivatives of *ortho*-carborane, the presence of intermolecular I⋯I dihalogen bonds inside them depends on the position of the substituents. In addition to 1,12-diiodo-*ortho*-carborane 1,12-I_2_-1,2-C_2_B_10_H_10_, which is characterized by the presence of strong intermolecular I⋯I dihalogen bonds (3.57 Å) [45], weak I⋯I dihalogen bonds (4.09 Å) were found in the 3,6-diiodo derivative 3,6-I_2_-1,2-C_2_B_10_H_10_ [50], while the 3,10-I_2_-1,2-C_2_B_10_H_10_ [49], 4,7-I_2_-1,2-C_2_B_10_H_10_ [51], and 9,12-I_2_-1,2-C_2_B_10_H_10_ [52] isomers do not form dihalogen bonds. Therefore, we were interested in studying the possibility of the formation of intermolecular I⋯I dihalogen bonds in triiodo-*ortho*-carboranes containing substituents in the three most electron-withdrawing positions of the caborane cage 1,2,3-I_3_-1,2-C_2_B_10_H_9_ and the three most electron-donating positions of 8,9,12-I_3_-1,2-C_2_B_10_H_9_.

The formation of 8,9,12-I_3_-1,2-C_2_B_10_H_9_ (**1**) was previously reported in the iodination of *ortho*-carborane with molecular iodine in acetic acid in the presence of a mixture of concentrated sulfuric and nitric acids [53]. We isolated the 8,9,12-triiodo derivative as a by-product of the reaction of *ortho*-carborane with iodine in dichloromethane in the presence of AlCl_3_ [54]. It should be noted that the unit cell parameters of 8,9,12-I_3_-1,2-C_2_B_10_H_9_ (**1**) have been reported [55]; however, its structure has not been yet solved.

**Figure 2:**
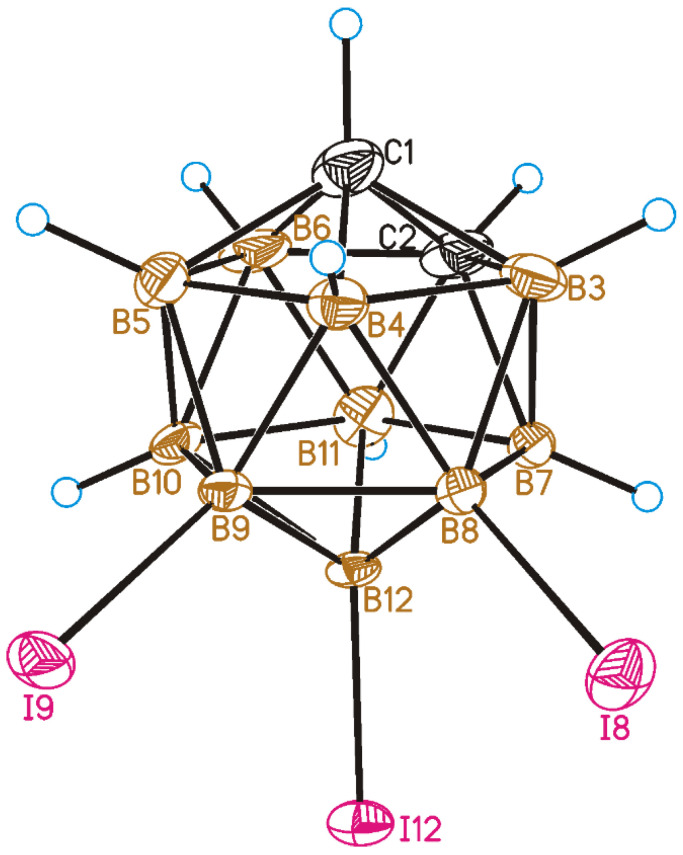
General view of 8,9,12-I_3_-1,2-C_2_B_10_H_9_ (**1**) showing atomic numbering. Thermal ellipsoids are given at 50% probability level.

The crystal structure of 8,9,12-I_3_-1,2-C_2_B_10_H_9_ was determined by single-crystal X-ray diffraction. A general view of **1** is presented in Figure 2. All the B-I distances in 8,9,12-I_3_-1,2-C_2_B_10_H_9_ are nearly equal (B8-I8 is 2.165(7)Å, B9-I9 is 2.160(7) Å, and B12-I12 is 2.160(7) Å) and are only slightly longer than the B-I distances in 8,9,10,12-I_4_-1,2-C_2_B_10_H_8_ (for which the average value is 2.151 Å) [46].

**Figure 3:**
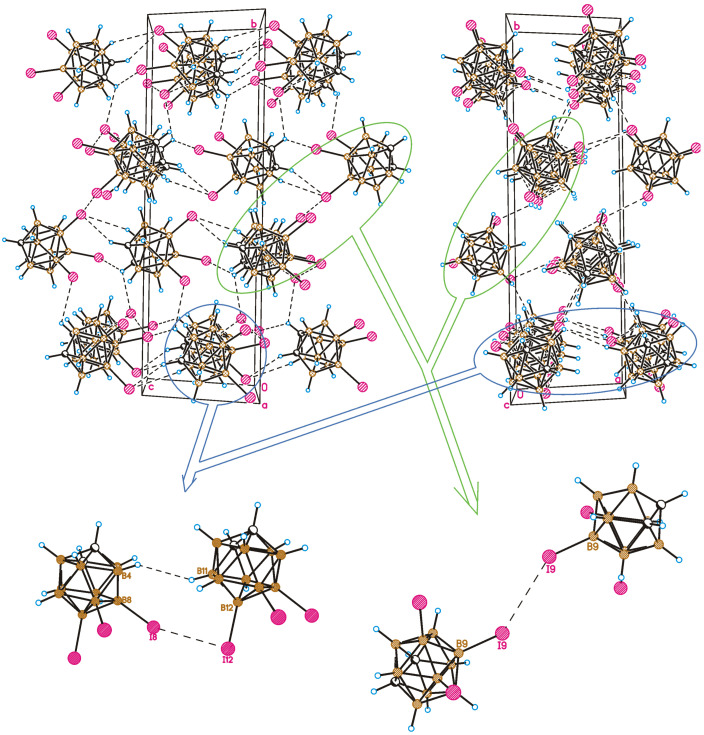
Crystal-packing fragment of 8,9,12-I_3_-1,2-C_2_B_10_H_9_. **Top-left**: view in the *bc* crystallographic plane; **top-right**: view in the *bc* crystallographic plane. Detailed view of type II weak halogen bonding (**bottom-left**) and type I halogen bonding (**bottom-right**).

A crystal-packing fragment of **1** is depicted in Figure 3. Only weak intermolecular interactions are observed in the crystal structure. From a formal point of view, four types of intermolecular interactions are observed in the crystal of **1**. Halogen atoms participate in both types (I and II) of halogen bonding, and I⋯H-C(B) hydrogen bonds as well as B-H⋯H-B contacts are formed. It should be noted that all intermolecular contacts except for one are somewhat longer than the sum of the van-der-Waals radii. For instance, the type II halogen bond is very weak (the I⋯I distance is 4.268(4) Å, the B8-I8⋯I12 angle is 130.2(3)°, and the B12-I12⋯I8 angle is 92.2(3)°) (Figure 3) and can only be regarded as a type II halogen bond formally. At the same time, the I9⋯I9 halogen bond of type I demonstrates an interhalogen distance (4.002(4) Å) shorter than the sum of the van-der-Waals radii (4.14 Å) [56]; however, halogen bonds of this type are usually relatively weak.

Therefore, it is impossible to choose one or two of the most important contacts that can be considered to be structure-forming. Interactions in the *bc* crystallographic plane are due to I⋯H-C(B) and H⋯H contacts, while in the crystallographic direction, *a*, molecules are linked mostly by I⋯I interactions. As a result, the crystal packing of 8,9,12-I_3_-1,2-C_2_B_10_H_9_ can be considered to be nearly isotropic.

It would be interesting to compare the crystal packing of 8,9,12-triiodo-*ortho*-carborane with that of its closest analog, 8,9,12-tribromo-*ortho*-carborane 8,9,12-Br_3_-1,2-C_2_B_10_H_9_ (**2**). Despite the fact that the bromination of *ortho*-carborane was first described as early as the mid-1960s [57], the chemistry of the bromo-derivatives of carborane has been studied to a much lesser extent compared to its iodo-derivatives due to the difficulty in isolating pure products. Recently, we published the synthesis and characterization of the 9,12-dibromo derivative of *ortho*-carborane [58]. Since the 8,9,12-tribromo derivative was one of the side-products of that reaction, we decided to increase the ratio of bromine to *ortho*-carborane (up to 3:1) and the reaction time. This allowed us to isolate the desired compound 8,9,12-Br_3_-1,2-C_2_B_10_H_9_ (**2**) at a 17% yield (see Section 3.3) It should be noted that the signal of the *CH* carborane groups of in the ^1^H NMR spectrum in CDCl_3_, which is a convenient indicator of the *CH*-acidity of carboranes [59,60], for compound **2** appears in a higher field at 3.87 ppm. compared to compound **1** (4.13 ppm). This indicates a lower acidity of the *CH*-carborane groups in the 8,9,12-tribromo derivative compared to the 8,9,12-triiodo derivative.

**Figure 4:**
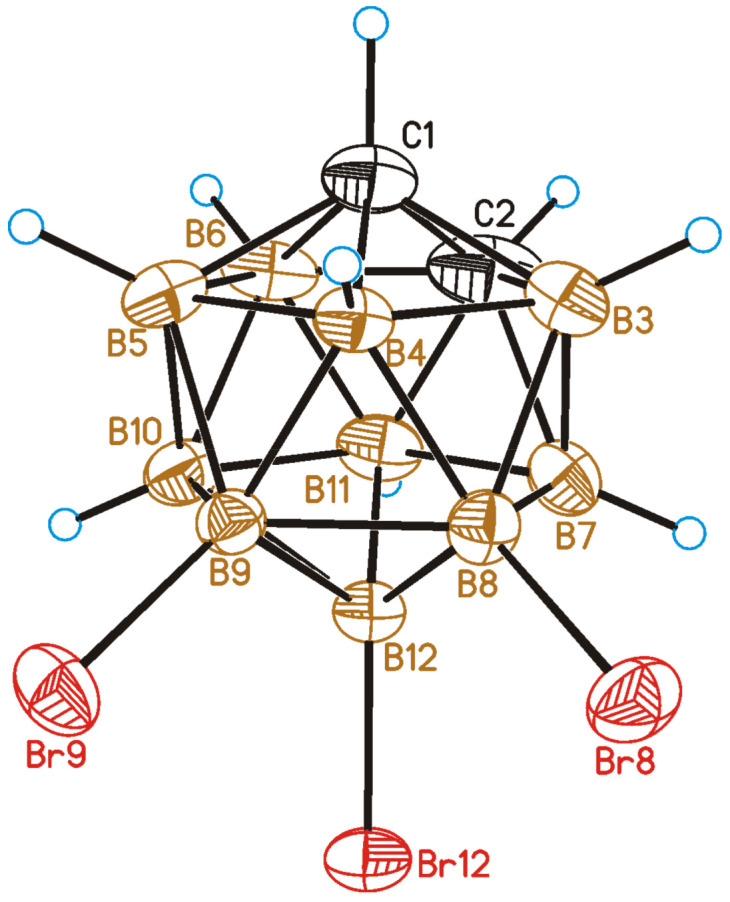
General view of 8,9,12-Br_3_-1,2-C_2_B_10_H_9_ (**2**) showing atomic numbering. Thermal ellipsoids are given at 50% probability level.

The crystal structure of 8,9,12-Br_3_-1,2-C_2_B_10_H_9_ was determined by single-crystal X-ray diffraction. A general view of **2** is presented in Figure 4.

It should be noted that the structure of 8,9,12-tibromo-*ortho*-carborane was determined in 1966 [61] at room temperature. The quality of that experiment was evidently low, and the experiment itself mostly concentrated on the description of the compound’s molecular geometry. Therefore, in the present study, we redetermined its structure at a low temperature (120 K), focusing on both its molecular structure and, especially, crystal-packing properties. Prior to the description of its crystal structure and comparison with that of **1**, it is interesting to mention some other studied bromo- and iodo-derivatives of *ortho*-carborane. For instance, the crystal structures of 1,2-Me_2_-8,9,10,12-I_4_-1,2-C_2_B_10_H_6_ [47] and 1,2-Me_2_-8,9,10,12-Br_4_-1,2-C_2_B_10_H_6_ [62] are isostructural. At the same time, the crystal structures of 1,12-I_2_-1,2-C_2_B_10_H_10_ [45] and 1,12-Br_2_-C_2_B_10_H_10_ [42] do not show any similarity. Only partial similarity in terms of crystal packing was observed for 9,12-I_2_-1,2-C_2_B_10_H_10_ [52] and 9,12-Br_2_-1,2-C_2_B_10_H_10_ [58]; however, the latter appeared to be isostructural to its chloro analog 9,12-Cl_2_-1,2-C_2_B_10_H_10_ [63] (see Figure S10 in SI).

**Figure 5:**
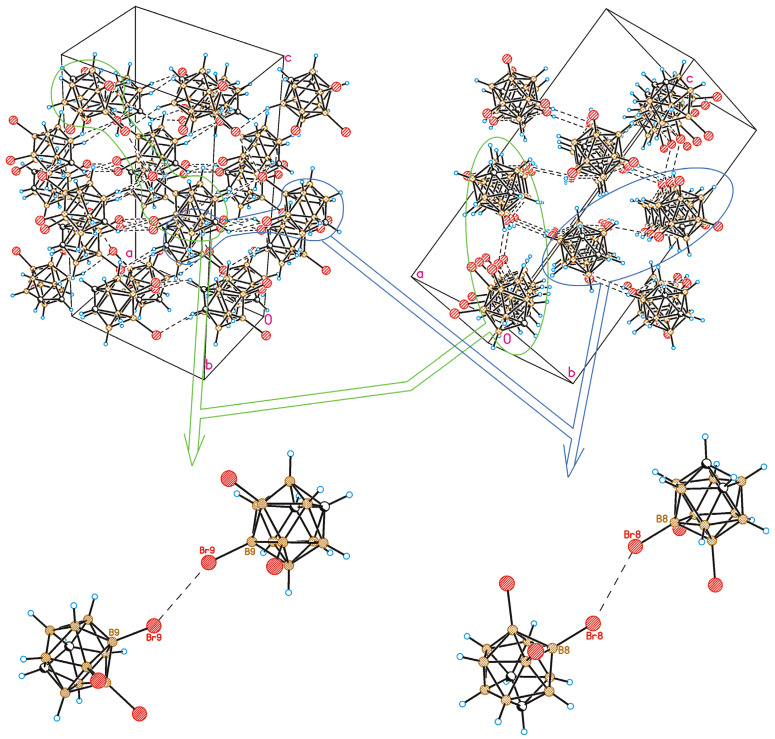
Crystal-packing fragment of 8,9,12-Br_3_-1,2-C_2_B_10_H_9_. Top-left: view down [1,−1,0] direction; top-right: view down [0,1,1] direction. Bottom: detailed view of type I halogen bonding (Br9⋯Br9, left; Br8⋯Br8, right). Projections of crystal packing are chosen to be consistent with those in Figure 3 and Figure 7 (orientations of molecules are nearly the same).

**Figure 6:**
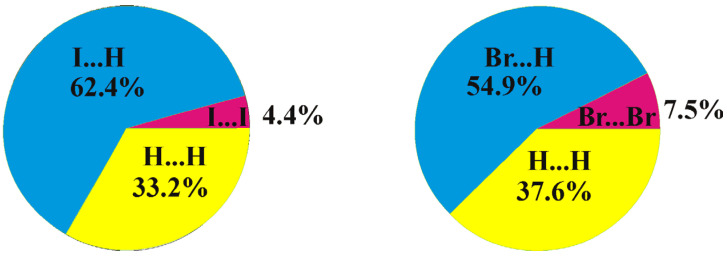
Distribution of intermolecular contacts in the crystal structures of 8,9,12-I_3_-1,2-C_2_B_10_H_9_ (**left**) and 8,9,12-Br_3_-1,2-C_2_B_10_H_9_ (**right**) as obtained using the Crystal Explorer program package [64].

A comparison of the crystal structures of **2** and **1** studied in this work demonstrates both similarities and differences. As in compound **1**, a Br9⋯Br9 halogen bond of type I is observed in the crystal structure of 8,9,12-Br_3_-1,2-C_2_B_10_H_9_ (the Br⋯Br distance is 3.586(2) Å, which is shorter than the sum of the van-der-Waals radii 3.79 Å) (Figure 5). At the same time, there are no type II halogen bonds; however, one more halogen bond of type I is found between Br8 atoms, wherein the Br⋯Br distance (3.969(2) Å) is somewhat longer than the sum of the van-der-Waals radii. As in compound **1**, all the other intermolecular interactions are Br⋯H-C(B) and H⋯H. The differences in the crystal-packing properties described above result in some redistribution of the contact types (Figure 6): the contribution of Hal⋯Hal contacts increases, which leads to a decrease in the number of Hal⋯H contacts and to an increase in H⋯H ones.

**Figure 7:**
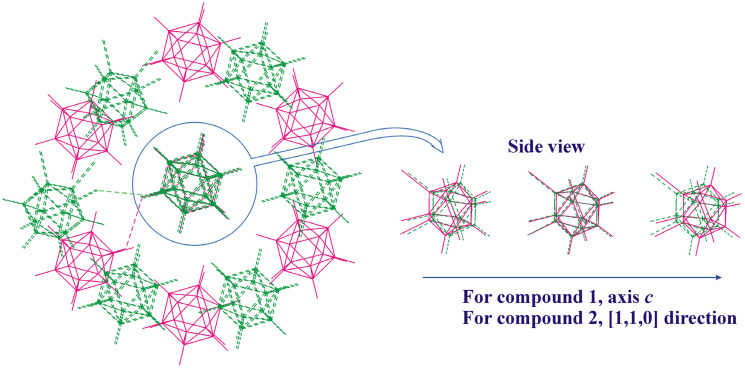
Superimposition of the closest environment of the crystal structure of compounds **1** (magenta) and **2** (green). The I9⋯I9 and Br9⋯Br9 halogen bonds (of type I) are shown by dashed lines on the left-side view.

The observed similarities and dissimilarities in the crystal packing of 8,9,12-I_3_-1,2-C_2_B_10_H_9_ and 8,9,12-Br_3_-1,2-C_2_B_10_H_9_ can be clearly seen in Figure 7. Similar C-H⋯I(Br)-bonded chains are formed in one direction, while in the perpendicular plane, the relative orientation of molecules is somewhat different.

**Figure 8:**
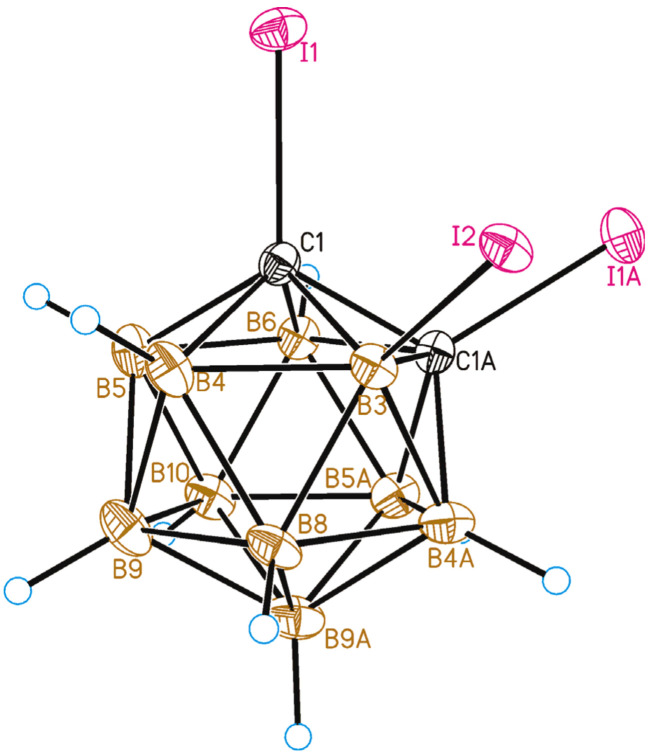
General view of 1,2,3-I_3_-1,2-C_2_B_10_H_9_ (**2**) showing atomic numbering. Thermal ellipsoids are given at 50% probability level.

The 1,2,3-isomer 1,2,3-I_3_-1,2-C_2_B_10_H_9_ (**3**) was prepared by the deprotonation of 3-iodo-*ortho*-carborane followed by a treatment of molecular iodine (see below). The crystal structure of 1,2,3-I_3_-1,2-C_2_B_10_H_9_ was determined by single-crystal X-ray diffraction. A general view of **3** is presented in Figure 8. The molecule in the crystal occupies a special position, as it is located at the two-fold symmetry axis. The C-I distances are the same (due to symmetry) and equal to 2.103(4) Å, while the B-I bond is somewhat longer at 2.160(5) Å. These lengths are slightly shorter than the C1-I1 (2.121(2) Å) and B12-I12 (2.179(2) Å) bonds in 1,12-I_2_-*closo*-C_2_B_10_H_10_ [45].

**Figure 9:**
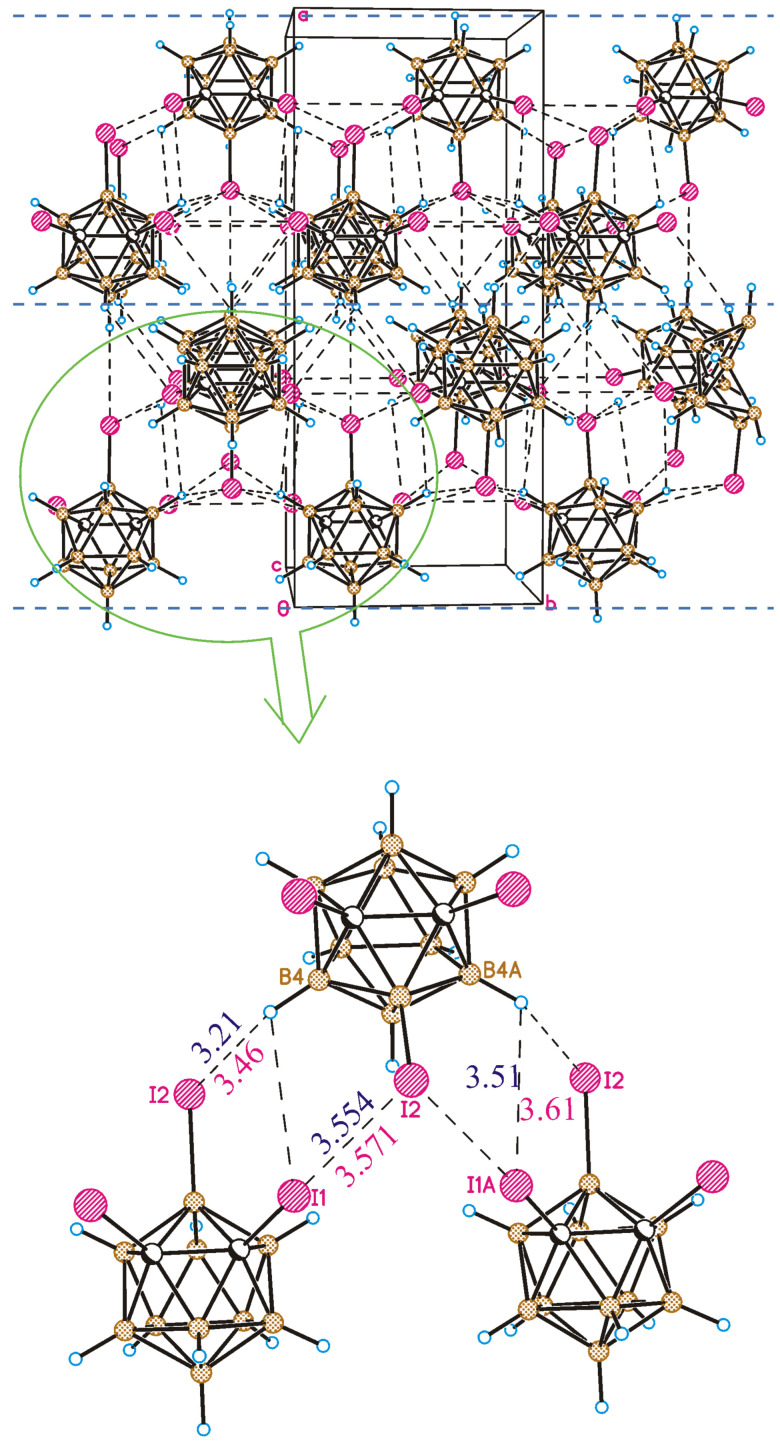
(**Top**) Crystal-packing fragment of 1,2,3-I_3_-1,2-C_2_B_10_H_9_. Blue, dashed lines separate halogen-bonded planes. (**Bottom**) Halogen-bonded trimer as a structure-forming unit of 1,2,3-I_3_-1,2-C_2_B_10_H_9_. Blue and red values correspond to the experimental and calculated I⋯I and H⋯I distances, respectively (given in Å).

Contrary to 8,9,12-I_3_-1,2-C_2_B_10_H_9_, the crystal packing of 1,2,3-I_3_-1,2-C_2_B_10_H_9_ is formed by halogen-bonded planes parallel to the *bc* crystallographic plane (Figure 9). In the planes, the I2 atom (attached to the boron atom) donates its lone pairs simultaneously to the σ-holes of both iodine atoms attached to the carbon atoms (the I1⋯I2 distance is 3.554(2) Å, the C1-I1⋯I2 angle is 169.2(2)°, and the B3-I2⋯I1 angle is 92.2(2)°). Therefore, the main structure-forming unit is the trimeric halogen-bonded associate.

In our recent study, we theoretically compared the dimer formation of 1,12- and 1,3-diiodo-*ortho*-carboranes [45]. According to our calculations, it appeared that both dimers are stabilized by a type II halogen bond and B-H⋯I hydrogen bonds. The role of the halogen bond is more pronounced in both dimers; however, in the 1,3-isomer, the halogen bond is weaker (but only by 2.5 kJ/mol), while the hydrogen bonds are stronger (in total by 0.4 kcal/mol). This means that the probability of the formation of a type II halogen bond in a real crystal of 1,3-I_2_-1,2-C_2_B_10_H_10_ is somewhat low. Nevertheless, it is formed and is a structure-forming interaction in the crystal structure of 1,2,3-I_3_-1,2-C_2_B_10_H_9_. Indeed, there are no H⋯H shortened contacts. The structure is additionally stabilized by a few B-H⋯I shortened contacts. However, some of them are formed between molecules already linked by halogen bonds. For a better understanding of the intermolecular connection in the trimers, we optimized its structure using density functional theory (DFT) at the PBE0/def2tzvp level followed by a topological analysis of the calculated electron density in terms of the “Atoms in Molecules” theory [65]. The intermolecular interaction energies were estimated from their correlation with the potential energy density at the bond critical point [66,67] using the AIMAll program [68].

**Figure 10:**
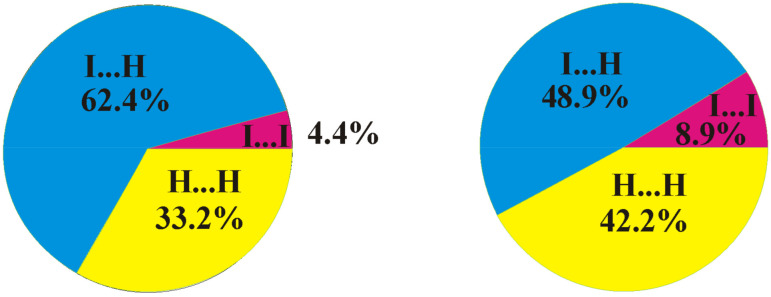
Distribution of intermolecular contacts in the crystal structures of 8,9,12-I_3_-1,2-C_2_B_10_H_9_ (**left**) and 1,2,3-I_3_-1,2-C_2_B_10_H_9_ (**right**).

This method of investigating structural details was successfully utilized in our recent studies on noncovalent interactions [69,70,71]. Good agreement was obtained between the calculated and experimental structures. The interhalogen distances are nearly the same (Figure 9), and the calculated angles C1-I1⋯I2 (168.2°) and B3-I2⋯I1 (90.5°) also strongly agree with the experiment. The H⋯I distances are somewhat shorter, as predicted by theory. According to the calculations, the energy of the halogen bond is equal to 8.8 kJ/mol, while the energies of the H4⋯I1 and H4⋯I2 contacts are 2.5 and 2.1 kJ/mol, respectively. Therefore, the attraction energy of each two molecules in the layer is equal to (8.8 + 2.5 + 2.1) 13.4 kJ/mol, while only weak B-H⋯I contacts are observed between layers. This allows us to consider the crystal packing of compound **3** as anisotropic unlike the 8,9,12-isomer. It is interesting to note that the crystal density of the latter is somewhat higher than that of the 1,2,3-isomer. This can be explained by the increased role of the I⋯I interactions (Figure 10). The presence of relatively strong I⋯I intermolecular interactions does not allow molecules to adjust their orientations to obtain closer packing.

The same reasons can be used to explain the higher density of water in comparison to ice, and have also been used to explain the differences in the crystal-packing density of polynitro compounds [72,73].

## 3. Materials and Methods

### 3.1. General Methods

The reactions were carried under an inert atmosphere. 3-Iodo-*ortho*-carborane was prepared according to a procedure from the literature [74]. 1,2-Dimethoxyethane was dried using standard procedures [75]. All other chemical reagents were purchased from Sigma Aldrich, Acros Organics, and ABCR and used without purification. The reaction progress was monitored by thin-layer chromatography (Merck F254 silica gel on aluminum plates) and visualized using 0.5% PdCl_2_ in 1% HCl in aq. MeOH (1:10). Acros Organics silica gel (0.060–0.200 mm) was used for column chromatography. The NMR spectra at 400 MHz (^1^H) and 128 MHz (^11^B) were recorded with Varian Inova 400 spectrometer. The residual signal of the NMR solvent relative to Me_4_Si was taken as the internal reference for ^1^H spectra. ^11^B NMR spectra were referenced using BF_3_·Et_2_O as external standard.

### 3.2. Preparation of 8,9,12-Triiodo-ortho-Carborane 8,9,12-I_3_-1,2-C_2_B_10_H_9_

8,9,12-I_3_-*ortho*-C_2_B_10_H_9_ was isolated as a by-product from the di-iodination reaction of *ortho*-carborane under standard conditions [51]. Iodine (3.553 g, 14.00 mmol) and anhydrous AlCl_3_ (0.400 g) were added to a solution of *ortho*-carborane (1.009 g, 7.00 mmol) in dichloromethane (30 mL) and heated under reflux for 8 h. Then, the reaction mixture was cooled and treated with a solution of Na_2_S_2_O_3_·5H_2_O (3.000 g) in water (50 mL). The organic phase was separated, and the aqueous fraction was extracted with dichloromethane (3 × 50 mL). The organic phases were combined, dried over Na_2_SO_4_, filtered, and concentrated under reduced pressure. The crude product was purified by column chromatography on silica using diethyl ether as eluent to yield 1.900 g (69%) of 9,12-I_2_-1,2-C_2_B_10_H_10_ and 0.102 g (3%) of 8,9,12-I_3_-1,2-C_2_B_10_H_9_ as white powders.

8,9,12-I_3_-1,2-C_2_B_10_H_9_: ^1^H NMR (CDCl_3_, ppm): 4.13 (2H, br s, C*H*_carb_), 3.8−2.0 (7H, br m, B*H*). ^11^B NMR (CDCl_3_, ppm): δ −6.1 (1B, d, *J* = 157 Hz), −11.5 (4B, s + d), −13.1 (2B, d, *J* = 171 Hz), −14.7 (1B, d, *J* = 220 Hz), −16.4 (1B, d, *J* = 220 Hz), and −17.2 (1B, s, *B(8)*).

### 3.3. Preparation of 8,9,12-Tribromo-ortho-Carborane 8,9,12-Br_3_-1,2-C_2_B_10_H_9_

Bromine (1.08 mL, 3.356 g, and 21.00 mmol) and anhydrous aluminum chloride (0.400 g) were added to a solution of *ortho*-carborane (1.009 mg and 7.00 mmol) in 1,2-dichloroethane (30 mL) and heated under reflux for 40 h. Then, the reaction mixture was cooled and treated with a solution of Na_2_S_2_O_3_·5H_2_O (5.000 g) in water (50 mL). The organic phase was separated, and the aqueous fraction was extracted with dichloromethane (3 × 50 mL). The organic phases were combined, dried over Na_2_SO_4_, filtered, and concentrated under reduced pressure. The crude product was purified by column chromatography on silica using chloroform as eluent to yield 0.450 g (17%) of 8,9,12-Br_3_-1,2-C_2_B_10_H_9_ as a white powder.

8,9,12-Br_3_-1,2-C_2_B_10_H_9_: ^1^H NMR (CDCl_3_, ppm): 3.87 (2H, br s, C*H*_carb_), 3.5−1.7 (7H, br m, B*H*). ^11^B NMR (CDCl_3_, ppm): 0.4 (2B, s, *B(9,12)*), −5.3 (1B, s, *B(8)*), −8.4 (1B, d, *J* = 161 Hz, *B(10)*), −13.9 (2B, d, *J* = 176 Hz, *B(4,7)*), −15.6 (2B, d, *J* = 178 Hz, *B(5,11)*), −17.1 (1B, d, *J* = 188 Hz, *B(3)*), and −20.4 (1B, d, *J* = 185 Hz, *B(6)*). ^13^C NMR (CDCl_3_, ppm): 45.5 (*C*H_carb_).

### 3.4. Preparation of 1,2,3-Triiodo-ortho-Carborane 1,2,3-I_3_-1,2-C_2_B_10_H_9_

A 2.25 M hexane solution of *n*-butyllithium (390 μL; 0.88 mmol) was added to a solution of 3-iodo-*ortho*-carborane (110 mg; 0.41 mmol) in 1,2-dimethoxyethane (10 mL) and stirred at room temperature for 1 h. Iodine (244 mg; 0.96 mmol) was added in one portion and the reaction mixture was stirred at room temperature overnight; then, it was treated with a solution of Na_2_S_2_O_3_·5H_2_O (250 mg) in water (50 mL). The organic phase was separated, and the aqueous fraction was extracted with dichloromethane (3 × 50 mL). The organic phases were combined, dried over Na_2_SO_4_, filtered, and concentrated under reduced pressure. The crude product was purified by column chromatography on silica using diethyl ether as eluent to yield 137 mg of mixture of 3-I-1,2-C_2_B_10_H_11_ and 1,2,3-I_3_-1,2-C_2_B_10_H_9_.

### 3.5. Single-Crystal X-ray Diffraction Study

Single-crystal X-ray diffraction experiments of **1**, **2,** and **3** (see Supplementary Materials) were carried out using SMART APEX2 CCD diffractometer (λ(Mo-Kα) = 0.71073 Å; graphite monochromator; ω-scans) at 120 K. Collected data were processed by the SAINT and SADABS programs incorporated into the APEX2 program package [76]. The structures were determined by direct methods and refined by the full-matrix-least-squares procedure against *F*^2^ in anisotropic approximation. The refinement was carried out with the SHELXTL program [77]. The CCDC numbers (2216663 for **1**, 2234154 for **2**, and 2216664 for **3**) contain the supplementary crystallographic data for this paper. These data can be obtained free of charge via www.ccdc.cam.ac.uk/data_request/cif.

Crystallographic data for 8,9,12-I_3_-1,2-C_2_B_10_H_9_ (**1**): C_2_H_9_B_10_I_3_ are monoclinic; space group *P*2_1_/*n*: *a* = 7.5776(6) Å, *b* = 24.0030(18) Å, *c* = 7.7535(6) Å, β = 109.487(2)°, *V* = 1329.46(18) Å^3^, and *Z* = 4, *M* = 521.89, *d*_cryst_ = 2.607 g·cm^−3^. *wR*2 = 0.0794 calculated on *F*^2^*_hkl_* for all 2611 independent reflections with 2*θ* < 52.1° (*GOF* = 1.117, *R* = 0.0343 calculated on *F_hkl_* for 2331 reflections with *I* > 2*σ*(*I*)).

Crystallographic data for 8,9,12-Br_3_-1,2-C_2_B_10_H_9_ (**2**): C_2_H_9_B_10_Br_3_ are monoclinic; space group *C*2/*c*: *a* = 12.1453(6) Å, *b* = 8.4794(5) Å, *c* = 23.0632(11) Å, β = 90.089(2)°, *V* = 2375.2(2) Å^3^, *Z* = 8, *M* = 380.92, *d*_cryst_ = 2.131 g·cm^−3^. *wR*2 = 0.0797 calculated on *F*^2^*_hkl_* for all 2349 independent reflections with 2*θ* < 52.1° (*GOF* = 1.024, *R* = 0.0347 calculated on *F_hkl_* for 1908 reflections with *I* > 2*σ*(*I*)).

Crystallographic data for 1,2,3-I_3_-1,2-C_2_B_10_H_9_ for (**3**): C_2_H_9_B_10_I_3_ are orthorhombic; space group *Pnma*: *a* = 19.1157(8) Å, *b* = 8.0014(3) Å, *c* = 8.7287(4) Å, *V* = 1335.08(10) Å^3^, *Z* = 4, *M* = 521.89, *d*_cryst_ = 2.596 g·cm^−3^. *wR*2 = 0.0580 calculated on *F*^2^*_hkl_* for all 1739 independent reflections with 2*θ* < 56.2° (*GOF* = 1.143, *R* = 0.0224 calculated on *F_hkl_* for 1613 reflections with *I* > 2*σ*(*I*)).

### 3.6. Quantum Chemical Calculation

Quantum chemical optimization of halogen-bonded trimeric associate of 1,2,3-I_3_-1,2-C_2_B_10_H_9_ was carried out using the Gaussian program [78]. The initial geometry for optimization was taken from the X-ray data. Optimization was carried out using PBE0 functional and triple-zeta basis set def2tzvp. For better agreement with experimental geometry, calculation was carried out within polarizable continuum model (PCM) using SCRF keyword in the Gaussian program and highly polar water molecule. It has recently been shown that such a method of calculation results in better agreement of the geometry for noncovalent interactions [45,69].

## Data Availability

Crystallographic data for the structures of 8,9,12-I_3_-1,2-C_2_B_10_H_9_ (**1**), 8,9,12-Br_3_-1,2-C_2_B_10_H_9_ (**2**), and 1,2,3-I_3_-1,2-C_2_B_10_H_9_ (**3**) were deposited in the Cambridge Crystallographic Data Centre as supplementary publications CCDC 2216663 (for **1**), 2234154 (for **2**), and 2216664 (for **3**). The Supplementary Information contains crystallographic data for compounds **1**, **2,** and **3**.

## Supplementary Materials

The following supporting information can be downloaded at: https://www.mdpi.com/article/10.3390/molecules28020875/s1; Crystallographic data for compounds **1**, **2,** and **3**.
