# Supplementary material for: How the Position of Substitution Affects Intermolecular Bonding in Halogen Derivatives of Carboranes: Crystal Structures of 1,2,3- and 8,9,12-Triiodo- and 8,9,12-Tribromo ortho-Carboranes
Source: Molecules. 2023 Jan 15;28(2):875. doi: 10.3390/molecules28020875 (PMC9865681; doi:10.3390/molecules28020875)

checkCIF/PLATON report

Structure factors have been supplied for datablock(s) siv40

THIS REPORT IS FOR GUIDANCE ONLY. IF USED AS PART OF A REVIEW PROCEDURE FOR PUBLICATION, IT SHOULD NOT REPLACE THE EXPERTISE OF AN EXPERIENCED CRYSTALLOGRAPHIC REFEREE.

No syntax errors found. CIF dictionary Interpreting this report

Datablock: siv40

```
Bond precision:   C-C = 0.0110 A          Wavelength=0.71073

Cell:              a=7.5776(6)     b=24.0030(18)        c=7.7535(6)
                   alpha=90        beta=109.487(2)      gamma=90
Temperature:       120 K

                 Calculated                      Reported
Volume           1329.46(18)                     1329.46(18)
Space group      P 21/n                          P 21/n
Hall group       -P 2yn                          -P 2yn
Moiety formula   C2 H9 B10 I3                    C2 H9 B10 I3
Sum formula      C2 H9 B10 I3                    C2 H9 B10 I3
Mr               521.89                          521.89
Dx,g cm-3        2.608                           2.607
Z                4                               4
Mu (mm-1)        7.006                           7.006
F000             920.0                           920.0
F000'            914.59
h,k,lmax         9,29,9                          9,29,9
Nref             2629                            2611
Tmin,Tmax        0.436,0.657                     0.393,0.657
Tmin'            0.427

Correction method= # Reported T Limits: Tmin=0.393 Tmax=0.657
AbsCorr = MULTI-SCAN

Data completeness= 0.993            Theta(max)= 26.069

                                                  wR2(reflections)=
R(reflections)= 0.0343( 2331)                     0.0794( 2611)

S = 1.117                  Npar= 136
```

The following ALERTS were generated. Each ALERT has the format
test-name_ALERT_alert-type_alert-level.
Click on the hyperlinks for more details of the test.

Alert level C

```
PLAT250_ALERT_2_C Large U3/U1 Ratio for Average U(i,j) Tensor ....        2.1 Note
PLAT342_ALERT_3_C Low Bond Precision on  C-C Bonds ...............      0.011 Ang.
PLAT351_ALERT_3_C Long   C-H (X0.96,N1.08A)  C1       - H1       .       1.12 Ang.
PLAT351_ALERT_3_C Long   C-H (X0.96,N1.08A)  C2       - H2       .       1.12 Ang.
PLAT790_ALERT_4_C Centre of Gravity not Within Unit Cell: Resd.  #          1 Note
              C2 H9 B10 I3
PLAT906_ALERT_3_C Large K Value in the Analysis of Variance ......      3.251 Check
PLAT911_ALERT_3_C Missing FCF Refl Between Thmin & STh/L=    0.600         14 Report
PLAT971_ALERT_2_C Check Calcd Resid. Dens.  1.01Ang From I9              2.16 eA-3
PLAT975_ALERT_2_C Check Calcd Resid. Dens.  0.91Ang From C1      .       0.56 eA-3
PLAT976_ALERT_2_C Check Calcd Resid. Dens.  1.08Ang From C2      .      -0.55 eA-3
PLAT977_ALERT_2_C Check Negative Difference Density on H1        .      -0.66 eA-3
PLAT977_ALERT_2_C Check Negative Difference Density on H6        .      -0.56 eA-3
```

Alert level G

```
PLAT083_ALERT_2_G SHELXL Second Parameter in WGHT  Unusually Large      12.31 Why ?
PLAT343_ALERT_2_G Unusual sp?      Angle Range in Main Residue for         C1 Check
PLAT343_ALERT_2_G Unusual sp?      Angle Range in Main Residue for         C2 Check
PLAT367_ALERT_2_G Long?  C(sp?)-C(sp?) Bond  C1       - C2       .       1.64 Ang.
PLAT910_ALERT_3_G Missing # of FCF Reflection(s) Below Theta(Min).          1 Note
PLAT912_ALERT_4_G Missing # of FCF Reflections Above STh/L=  0.600          3 Note
PLAT913_ALERT_3_G Missing # of Very Strong Reflections in FCF ....          1 Note
PLAT933_ALERT_2_G Number of HKL-OMIT Records in Embedded .res File          6 Note
PLAT941_ALERT_3_G Average HKL Measurement Multiplicity ..........         3.4 Low
PLAT978_ALERT_2_G Number C-C Bonds with Positive Residual Density.          0 Info
```

0 **ALERT level A** = Most likely a serious problem - resolve or explain
0 **ALERT level B** = A potentially serious problem, consider carefully
12 **ALERT level C** = Check. Ensure it is not caused by an omission or oversight
10 **ALERT level G** = General information/check it is not something unexpected

0 ALERT type 1 CIF construction/syntax error, inconsistent or missing data
12 ALERT type 2 Indicator that the structure model may be wrong or deficient
8 ALERT type 3 Indicator that the structure quality may be low
2 ALERT type 4 Improvement, methodology, query or suggestion
0 ALERT type 5 Informative message, check

It is advisable to attempt to resolve as many as possible of the alerts in all categories. Often the minor alerts point to easily fixed oversights, errors and omissions in your CIF or refinement strategy, so attention to these fine details can be worthwhile. In order to resolve some of the more serious problems it may be necessary to carry out additional measurements or structure refinements. However, the purpose of your study may justify the reported deviations and the more serious of these should normally be commented upon in the discussion or experimental section of a paper or in the "special_details" fields of the CIF. checkCIF was carefully designed to identify outliers and unusual parameters, but every test has its limitations and alerts that are not important in a particular case may appear. Conversely, the absence of alerts does not guarantee there are no aspects of the results needing attention. It is up to the individual to critically assess their own results and, if necessary, seek expert advice.

Publication of your CIF in IUCr journals

A basic structural check has been run on your CIF. These basic checks will be run on all CIFs submitted for publication in IUCr journals (*Acta Crystallographica*, *Journal of Applied Crystallography*, *Journal of Synchrotron Radiation*); however, if you intend to submit to *Acta Crystallographica Section C* or *E* or *IUCrData*, you should make sure that full publication checks are run on the final version of your CIF prior to submission.

Publication of your CIF in other journals

Please refer to the *Notes for Authors* of the relevant journal for any special instructions relating to CIF submission.

PLATON version of 12/09/2022; check.def file version of 09/08/2022

Datablock siv40 - ellipsoid plot

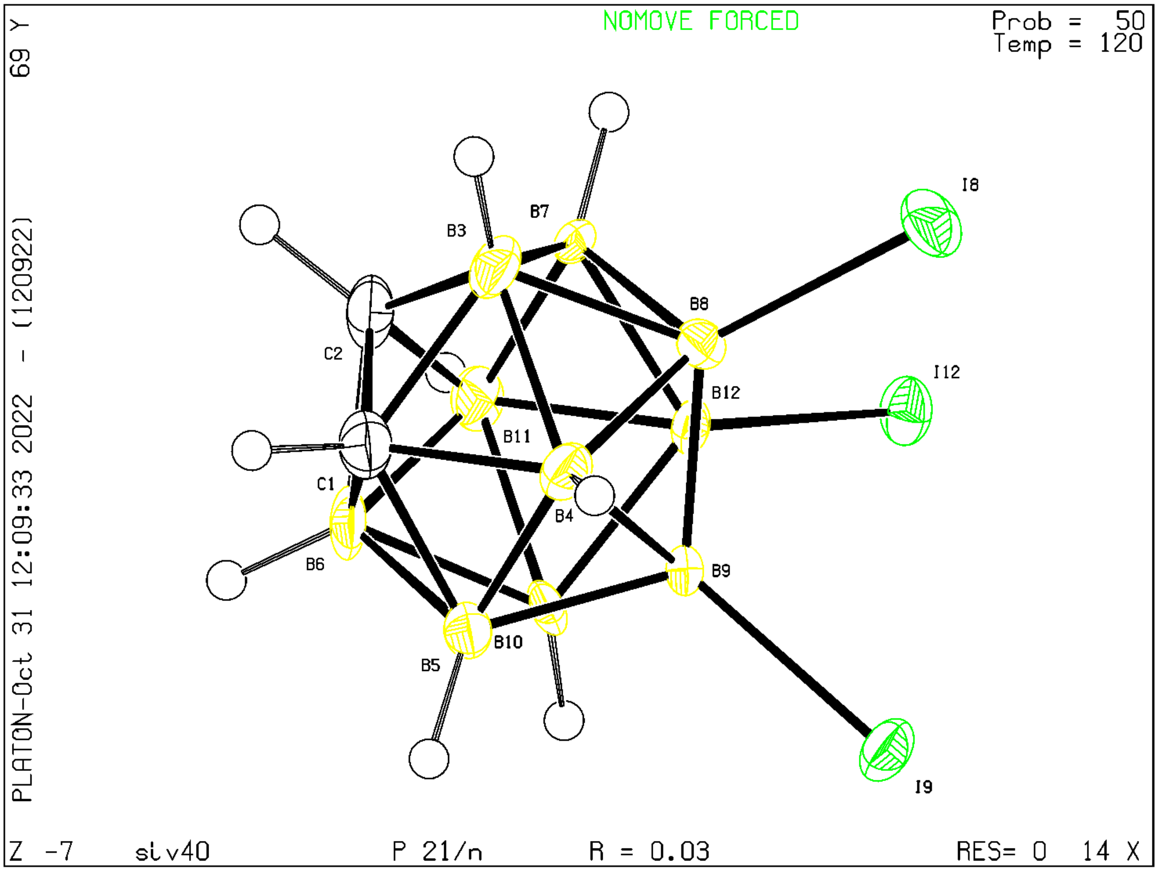

Supplement: Supplementary file 1 [file molecules-28-00875-s001.zip › checkcif_40.pdf]
