# Supplementary material for: How the Position of Substitution Affects Intermolecular Bonding in Halogen Derivatives of Carboranes: Crystal Structures of 1,2,3- and 8,9,12-Triiodo- and 8,9,12-Tribromo ortho-Carboranes
Source: Molecules. 2023 Jan 15;28(2):875. doi: 10.3390/molecules28020875 (PMC9865681; doi:10.3390/molecules28020875)

checkCIF/PLATON report

Structure factors have been supplied for datablock(s) siv75

THIS REPORT IS FOR GUIDANCE ONLY. IF USED AS PART OF A REVIEW PROCEDURE FOR PUBLICATION, IT SHOULD NOT REPLACE THE EXPERTISE OF AN EXPERIENCED CRYSTALLOGRAPHIC REFEREE.

No syntax errors found.   CIF dictionary   Interpreting this report

Datablock: siv75

```
Bond precision:   C-C = 0.0080 A          Wavelength=0.71073

Cell:              a=12.1453(6)     b=8.4794(5)        c=23.0632(11)
                   alpha=90         beta=90.089(2)     gamma=90
Temperature:       120 K

                  Calculated                      Reported
Volume            2375.2(2)                       2375.2(2)
Space group       C 2/c                           C 2/c
Hall group        -C 2yc                          -C 2yc
Moiety formula    C2 H9 B10 Br3                   C2 H9 B10 Br3
Sum formula       C2 H9 B10 Br3                   C2 H9 B10 Br3
Mr                380.89                          380.92
Dx,g cm-3         2.130                           2.131
Z                 8                               8
Mu (mm-1)         10.136                          10.136
F000              1408.0                          1408.0
F000'             1402.15
h,k,lmax          14,10,28                        14,10,28
Nref              2349                            2349
Tmin,Tmax         0.308,0.444                     0.276,0.444
Tmin'             0.233

Correction method= # Reported T Limits: Tmin=0.276 Tmax=0.444
AbsCorr = MULTI-SCAN

Data completeness= 1.000           Theta(max)= 26.026

R(reflections)= 0.0347( 1908)                          wR2(reflections)=
                                                       0.0797( 2349)
S = 1.024                  Npar= 136
```

```
The following ALERTS were generated. Each ALERT has the format
       test-name_ALERT_alert-type_alert-level.
Click on the hyperlinks for more details of the test.
```

Alert level C

```
PLAT230_ALERT_2_C Hirshfeld Test Diff for    C2       --B6       .        6.6 s.u.
PLAT241_ALERT_2_C High   'MainMol' Ueq as Compared to Neighbors of         C2 Check
PLAT341_ALERT_3_C Low Bond Precision on  C-C Bonds ...............      0.008 Ang.
PLAT351_ALERT_3_C Long   C-H (X0.96,N1.08A)  C1       - H1       .       1.12 Ang.
PLAT351_ALERT_3_C Long   C-H (X0.96,N1.08A)  C2       - H2       .       1.12 Ang.
```

Alert level G

```
PLAT343_ALERT_2_G Unusual sp?      Angle Range in Main Residue for         C1 Check
PLAT343_ALERT_2_G Unusual sp?      Angle Range in Main Residue for         C2 Check
PLAT367_ALERT_2_G Long?  C(sp?)-C(sp?) Bond  C1       - C2       .       1.66 Ang.
PLAT434_ALERT_2_G Short Inter HL..HL Contact Br9      ..Br9      .       3.59 Ang.
                                           -x,y,1/2-z  =      2_555 Check
PLAT910_ALERT_3_G Missing # of FCF Reflection(s) Below Theta(Min).          1 Note
PLAT933_ALERT_2_G Number of HKL-OMIT Records in Embedded .res File          1 Note
PLAT941_ALERT_3_G Average HKL Measurement Multiplicity ...........        4.4 Low
PLAT978_ALERT_2_G Number C-C Bonds with Positive Residual Density.          0 Info
```

```
   0 ALERT level A = Most likely a serious problem - resolve or explain
   0 ALERT level B = A potentially serious problem, consider carefully
   5 ALERT level C = Check. Ensure it is not caused by an omission or oversight
   8 ALERT level G = General information/check it is not something unexpected

   0 ALERT type 1 CIF construction/syntax error, inconsistent or missing data
   8 ALERT type 2 Indicator that the structure model may be wrong or deficient
   5 ALERT type 3 Indicator that the structure quality may be low
   0 ALERT type 4 Improvement, methodology, query or suggestion
   0 ALERT type 5 Informative message, check
```

Validation response form

Please find below a validation response form (VRF) that can be filled in and pasted into your CIF.

```
# start Validation Reply Form
_vrf_PLAT230_siv75
;
PROBLEM: Hirshfeld Test Diff for    C2       --B6       .        6.6 s.u.
RESPONSE: ...
;
_vrf_PLAT241_siv75
;
PROBLEM: High   'MainMol' Ueq as Compared to Neighbors of         C2 Check
RESPONSE: ...
;
_vrf_PLAT341_siv75
;
PROBLEM: Low Bond Precision on  C-C Bonds ..............      0.008 Ang.
```

```
RESPONSE: ...
;
_vrf_PLAT351_siv75
;
PROBLEM: Long   C-H (X0.96,N1.08A)  C1       - H1       .       1.12 Ang.
RESPONSE: ...
;
# end Validation Reply Form
```

It is advisable to attempt to resolve as many as possible of the alerts in all categories. Often the minor alerts point to easily fixed oversights, errors and omissions in your CIF or refinement strategy, so attention to these fine details can be worthwhile. In order to resolve some of the more serious problems it may be necessary to carry out additional measurements or structure refinements. However, the purpose of your study may justify the reported deviations and the more serious of these should normally be commented upon in the discussion or experimental section of a paper or in the "special_details" fields of the CIF. checkCIF was carefully designed to identify outliers and unusual parameters, but every test has its limitations and alerts that are not important in a particular case may appear. Conversely, the absence of alerts does not guarantee there are no aspects of the results needing attention. It is up to the individual to critically assess their own results and, if necessary, seek expert advice.

Publication of your CIF in IUCr journals

A basic structural check has been run on your CIF. These basic checks will be run on all CIFs submitted for publication in IUCr journals (*Acta Crystallographica*, *Journal of Applied Crystallography*, *Journal of Synchrotron Radiation*); however, if you intend to submit to *Acta Crystallographica Section C* or *E* or *IUCrData*, you should make sure that full publication checks are run on the final version of your CIF prior to submission.

Publication of your CIF in other journals

Please refer to the *Notes for Authors* of the relevant journal for any special instructions relating to CIF submission.

PLATON version of 28/11/2022; check.def file version of 28/11/2022

Datablock siv75 - ellipsoid plot

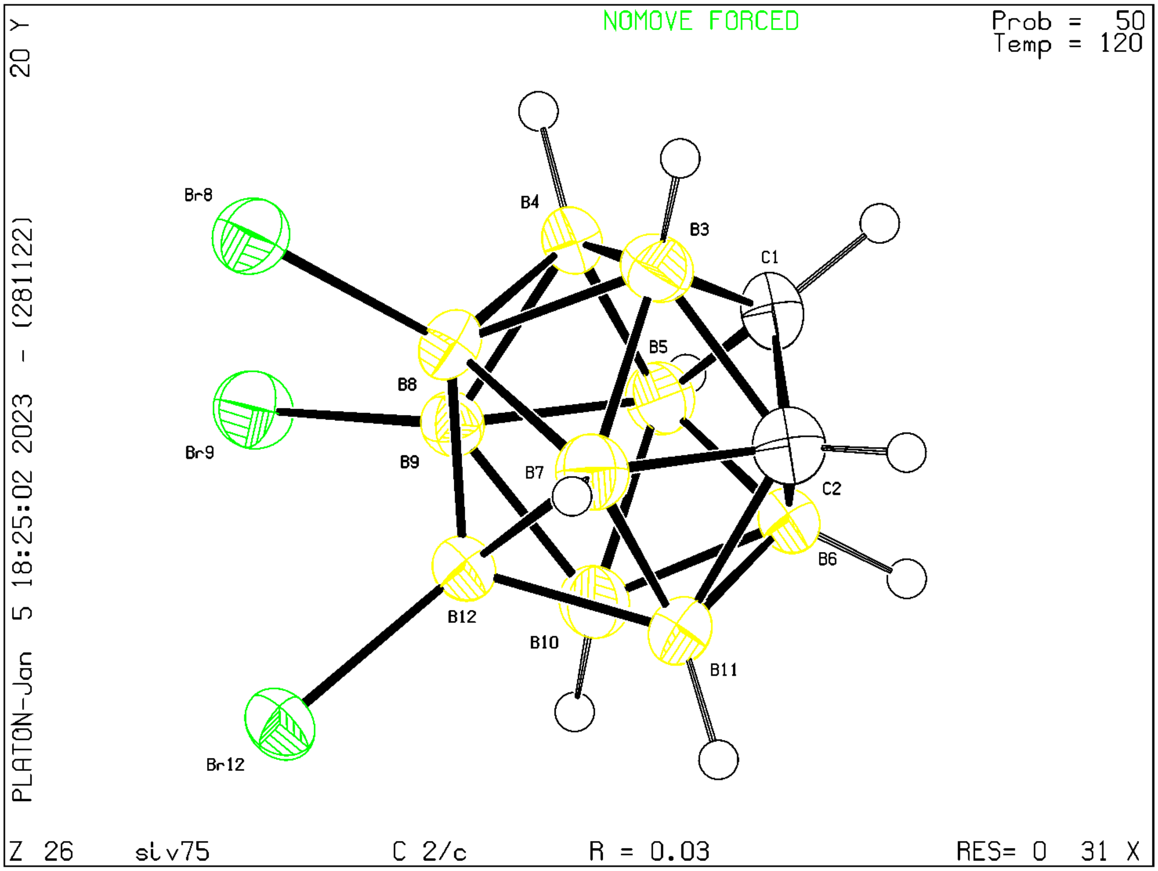

Supplement: Supplementary file 1 [file molecules-28-00875-s001.zip › checkcif_75.pdf]
