# Supplementary material for: How the Position of Substitution Affects Intermolecular Bonding in Halogen Derivatives of Carboranes: Crystal Structures of 1,2,3- and 8,9,12-Triiodo- and 8,9,12-Tribromo ortho-Carboranes
Source: Molecules. 2023 Jan 15;28(2):875. doi: 10.3390/molecules28020875 (PMC9865681; doi:10.3390/molecules28020875)
Supplement: Supplementary file 1 [file molecules-28-00875-s001.zip › Supplementary Materials.pdf]

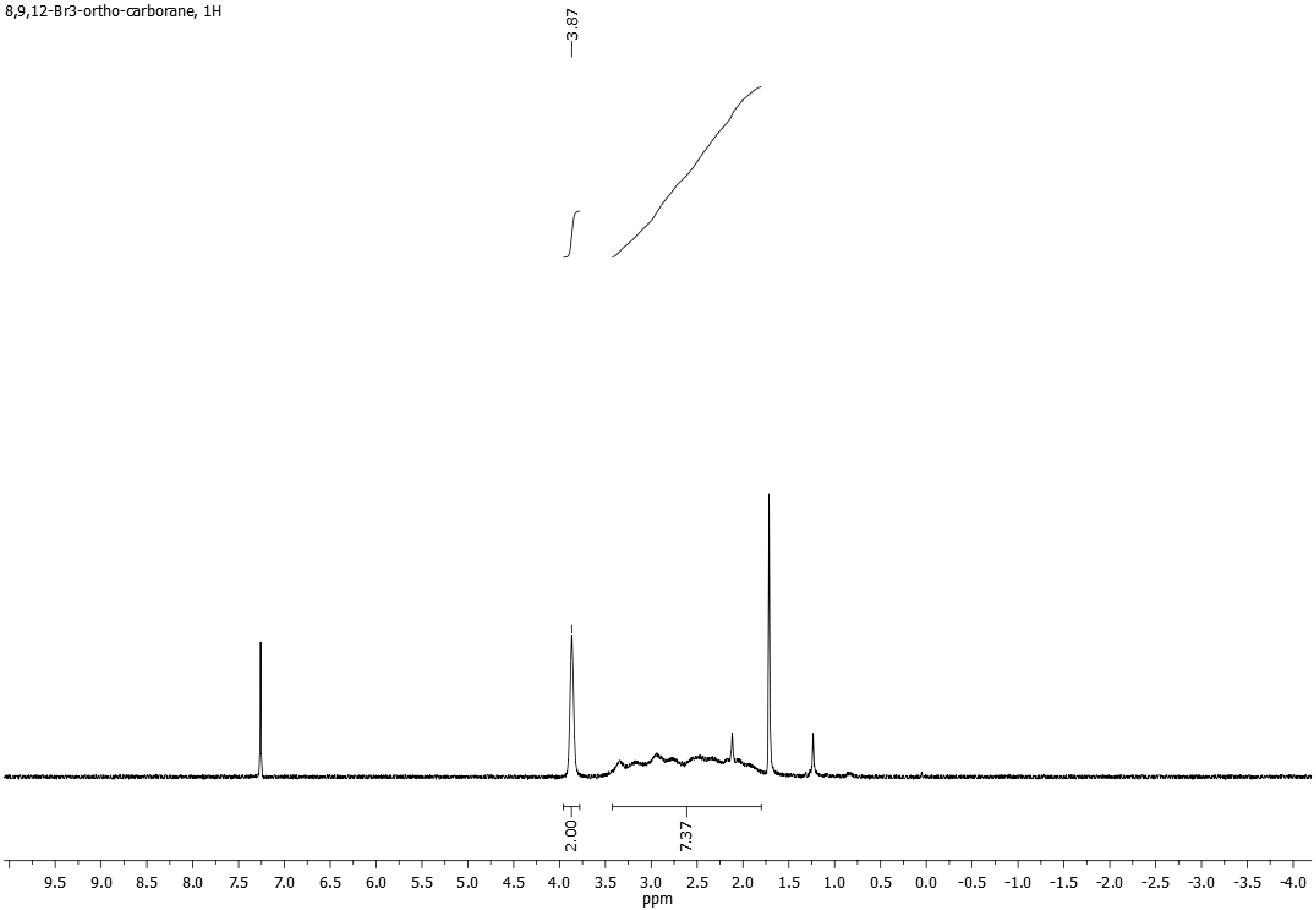

Figure S1. ^{1}H NMR spectrum of 8,9,12-Br_3-*ortho*-carborane.

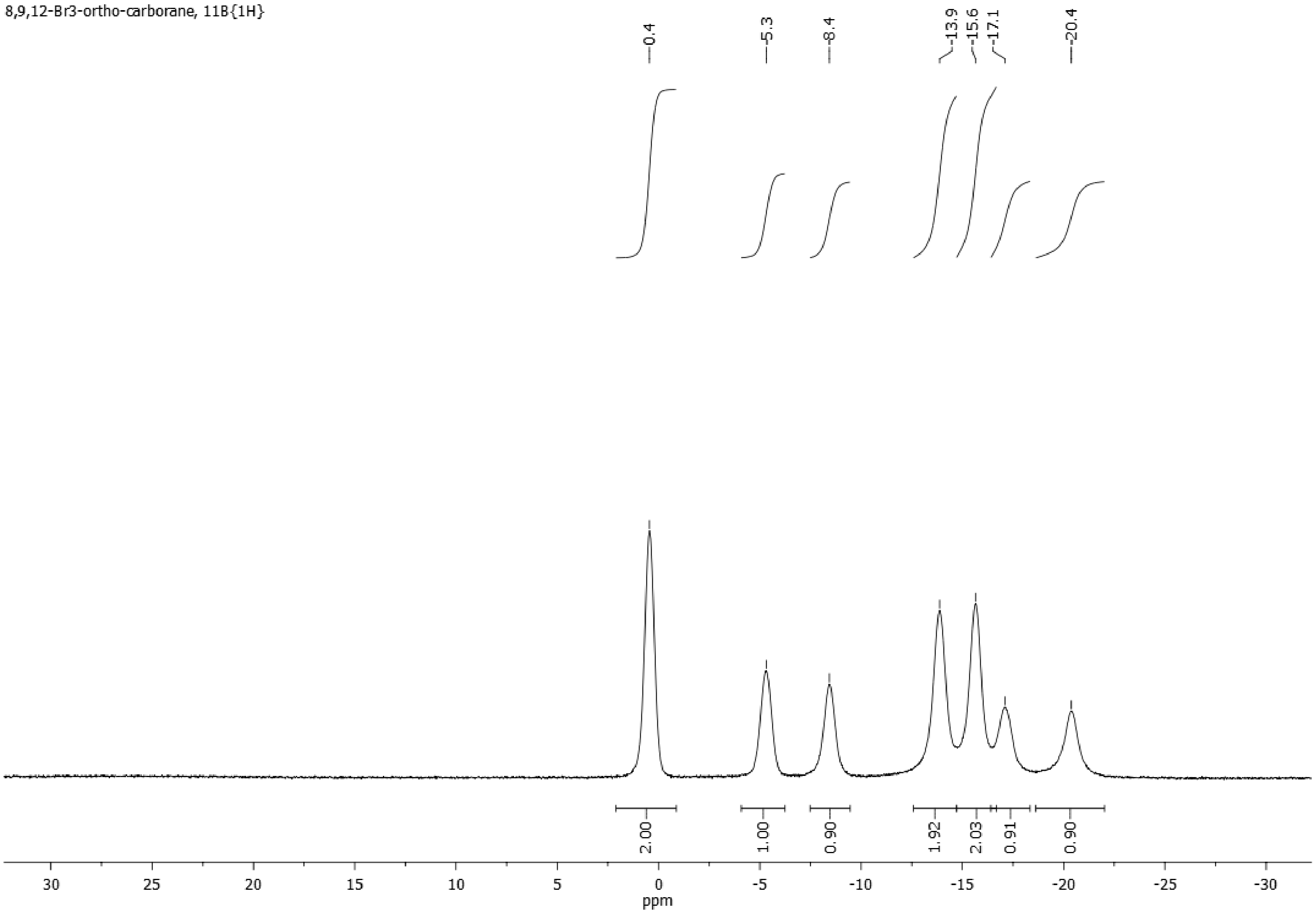

Figure S2. $^{11}B\{^{1}H\}$ NMR spectrum of 8,9,12-Br_3-*ortho*-carborane.

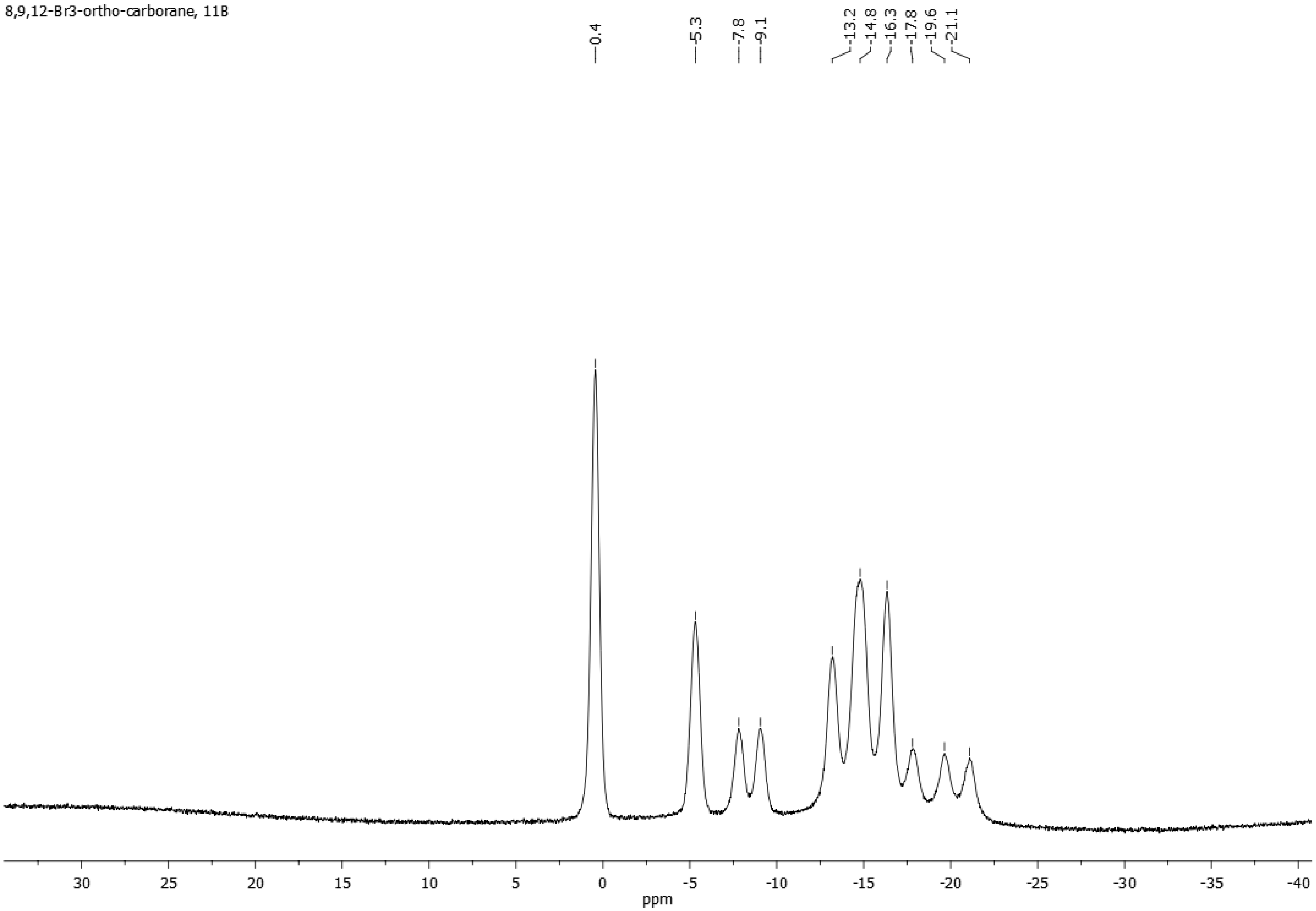

Figure S3. ^{11}B NMR spectrum of 8,9,12-Br_3-*ortho*-carborane.

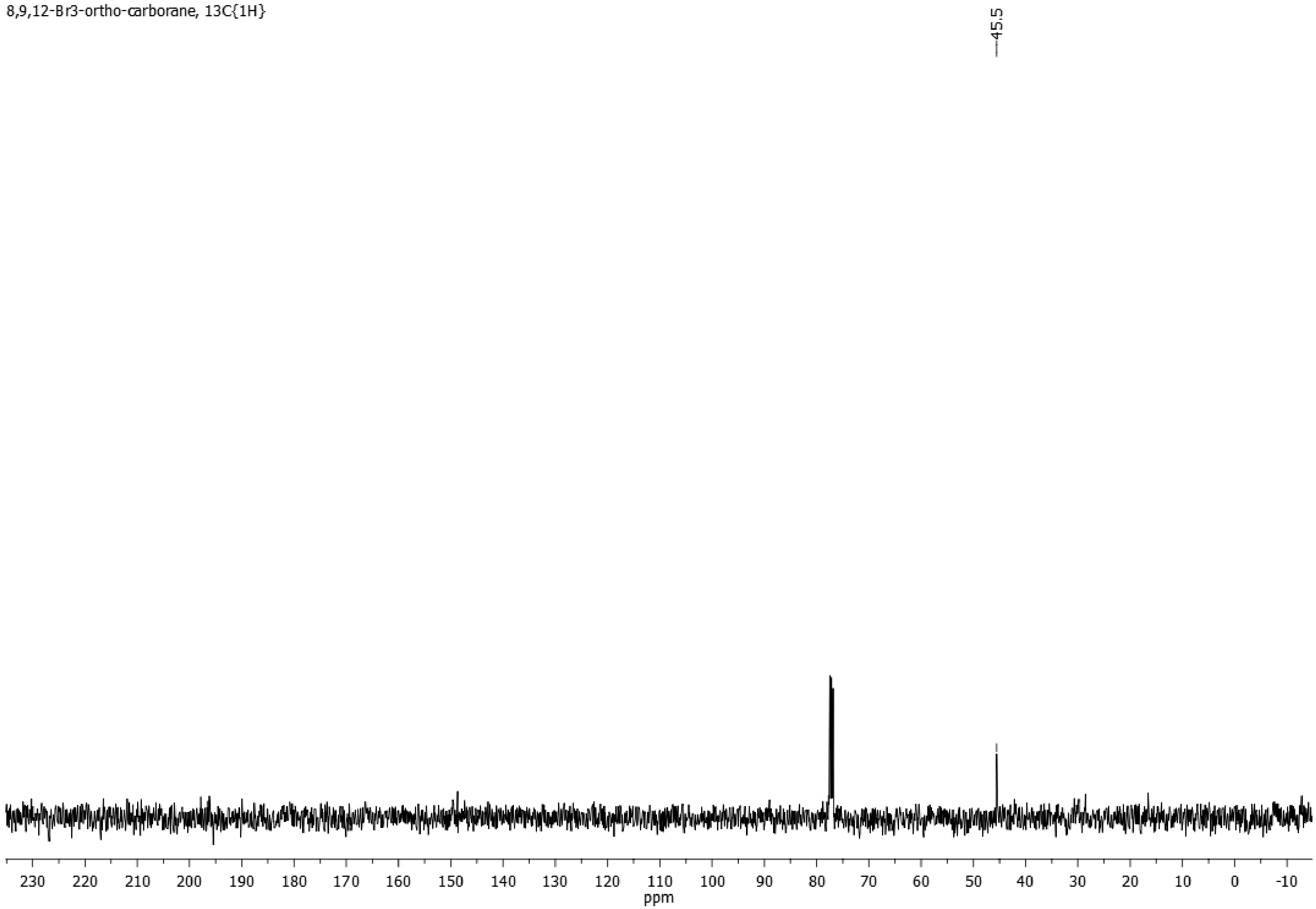

Figure S4. ^{13}C NMR spectrum of 8,9,12-Br_3-*ortho*-carborane.

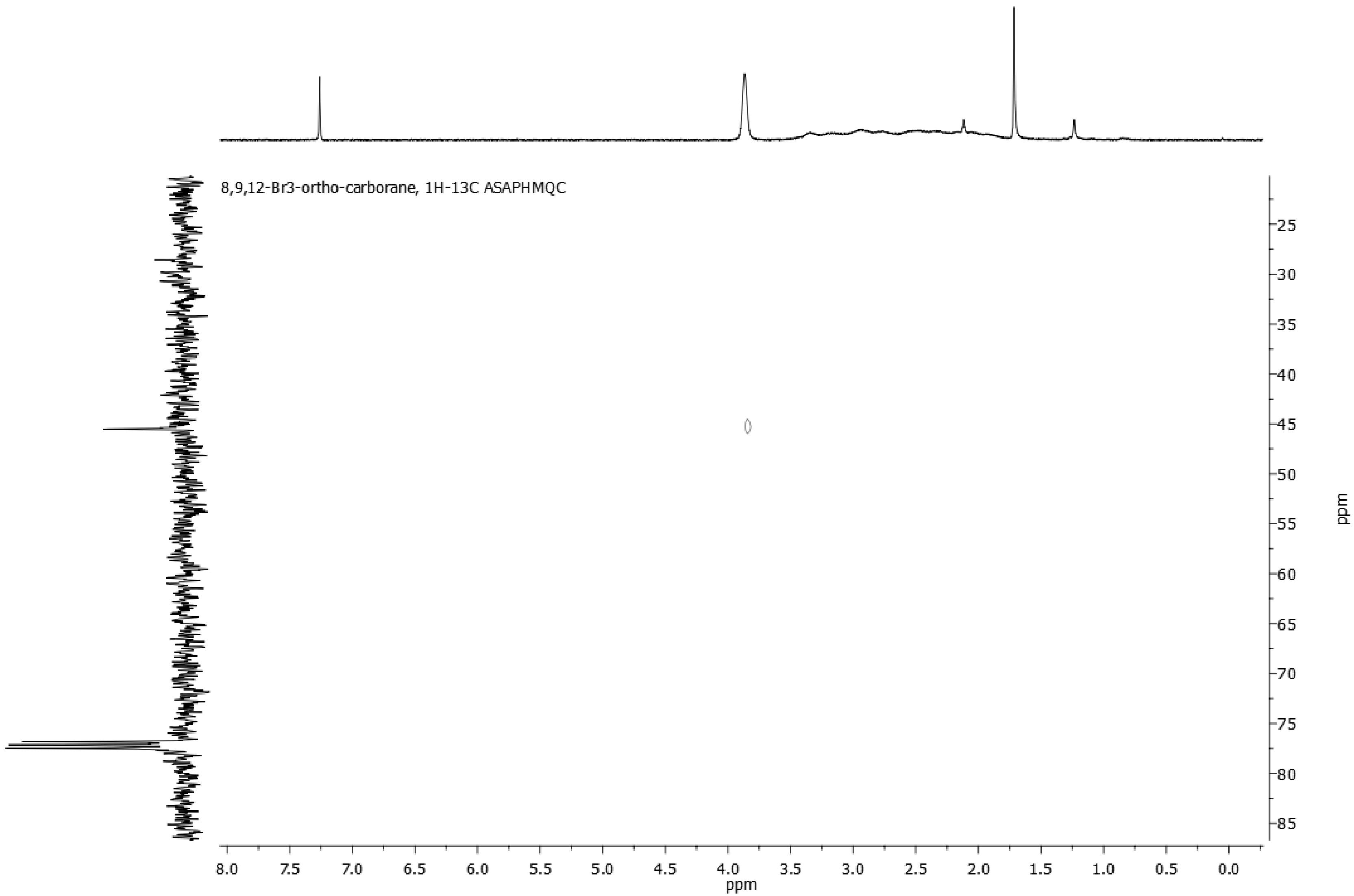

Figure S5. ^{1}H-^{13}C ASAPHMQC NMR spectrum of 8,9,12-Br_3-*ortho*-carborane.

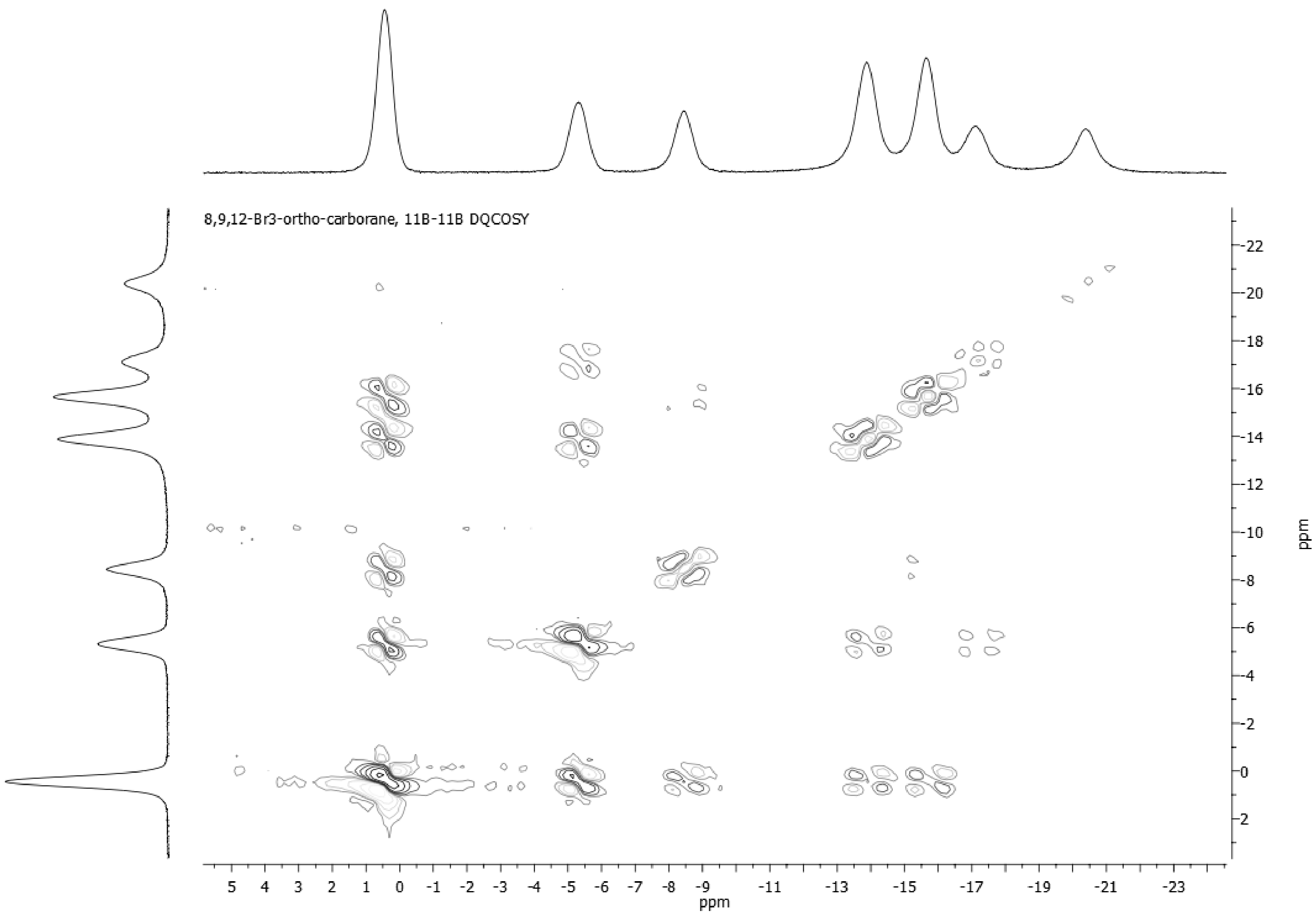

Figure S6. ^{11}B-^{11}B DQCOSY NMR spectrum of 8,9,12-Br_3-*ortho*-carborane.

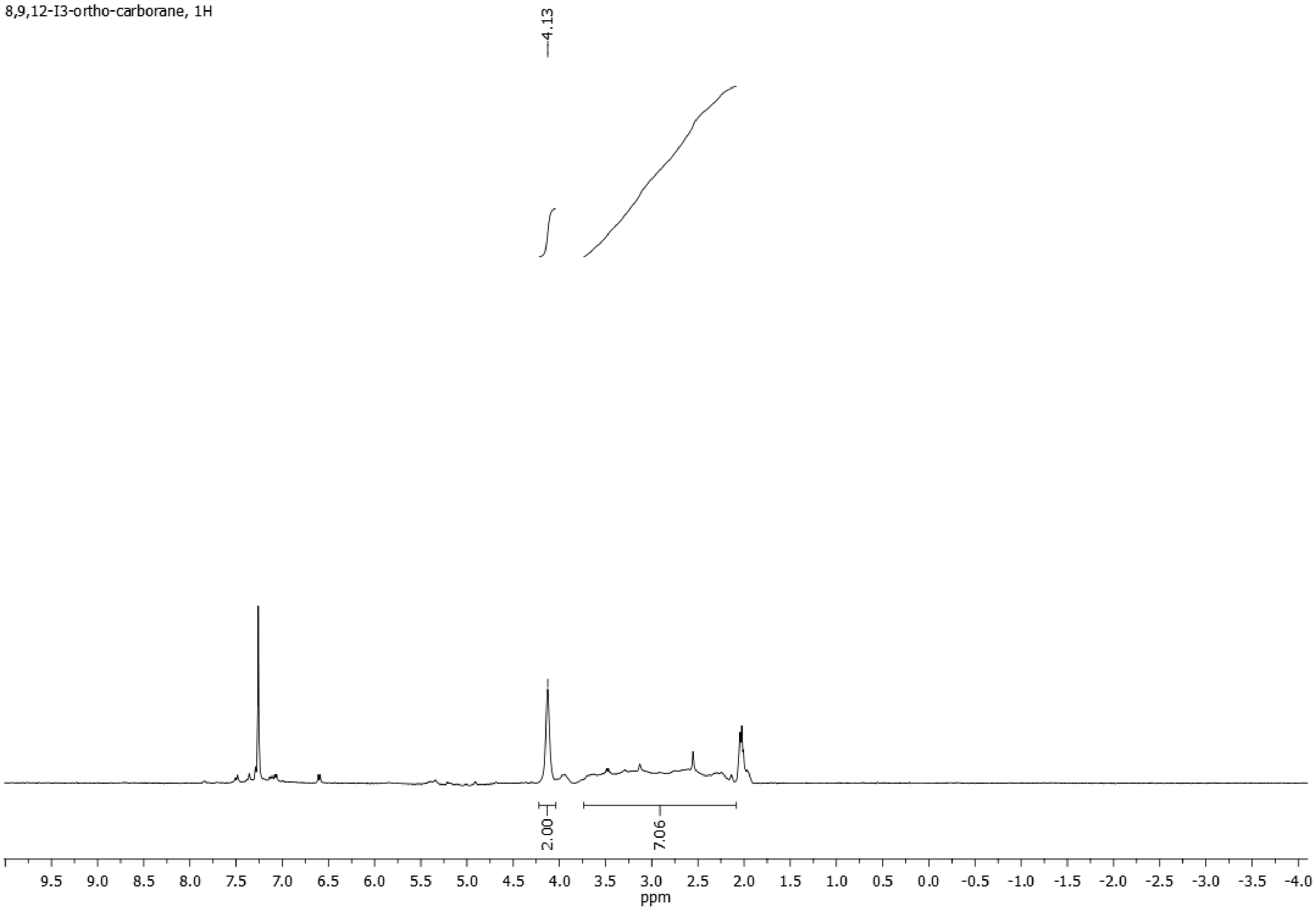

Figure S7. ^{1}H NMR spectrum of 8,9,12-I_3-*ortho*-carborane.

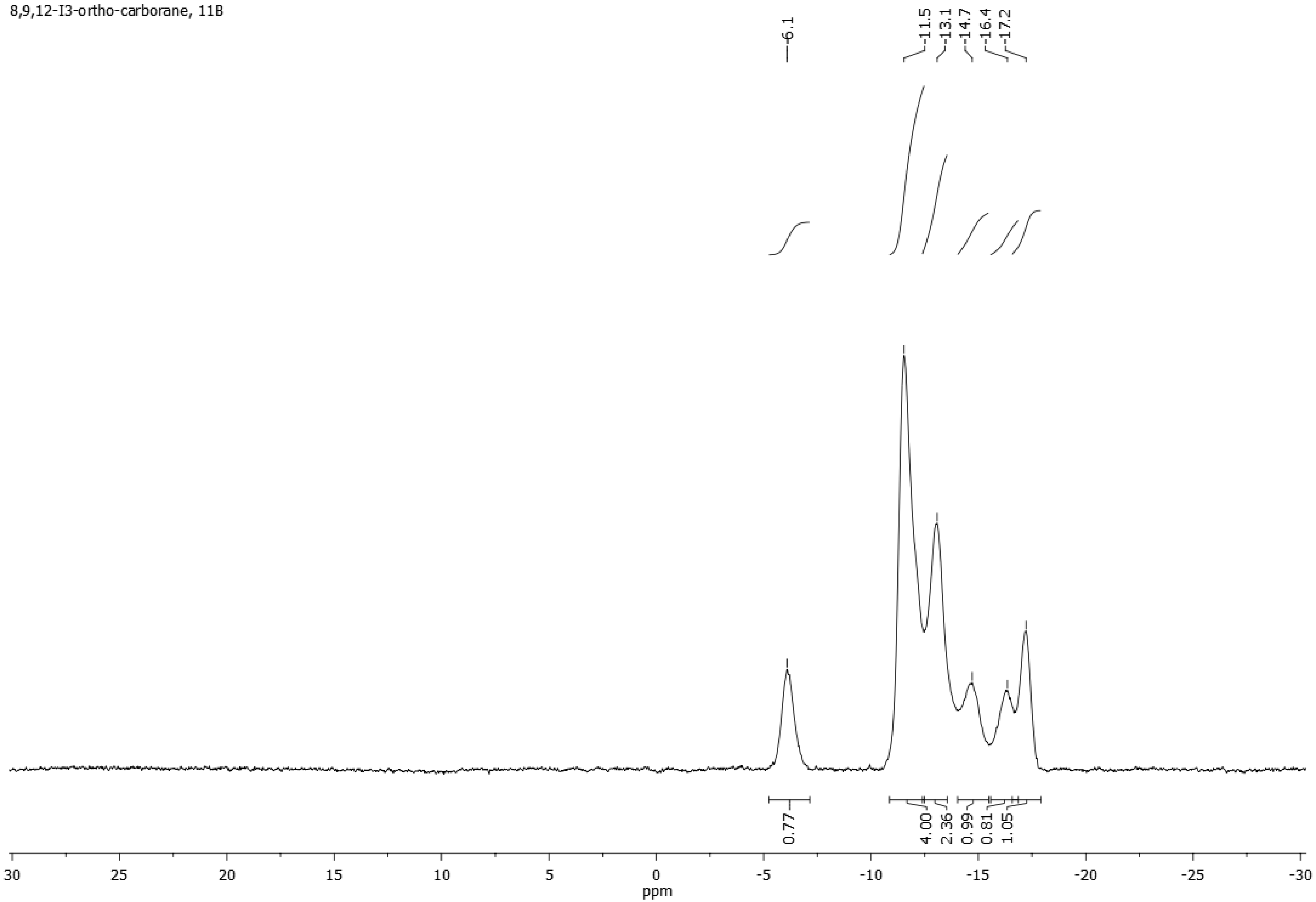

Figure S8. ^{11}H NMR spectrum of 8,9,12-I_3-*ortho*-carborane.

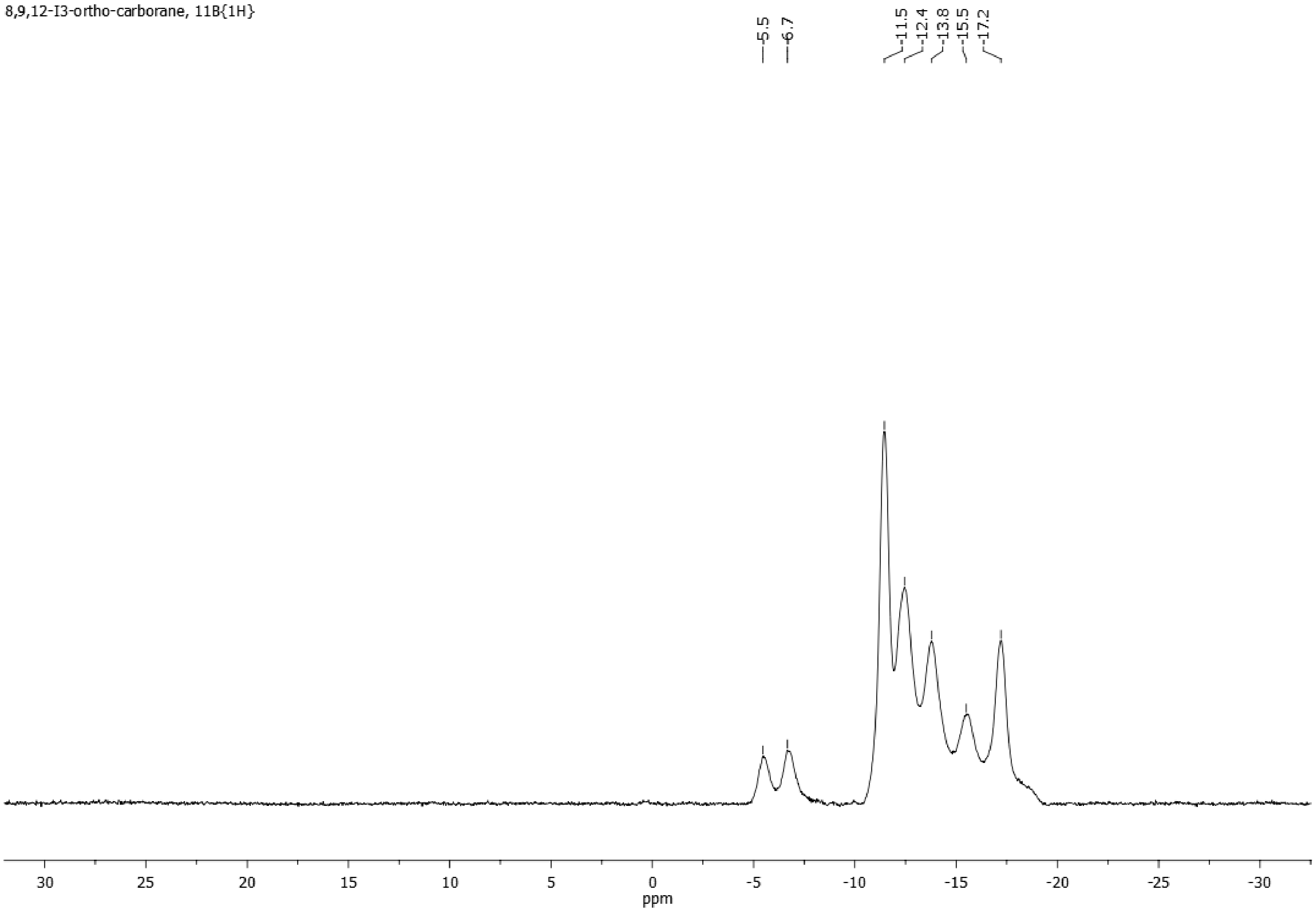

Figure S9. $^{11}B\{^{1}H\}$ NMR spectrum of 8,9,12-I_3-*ortho*-carborane.

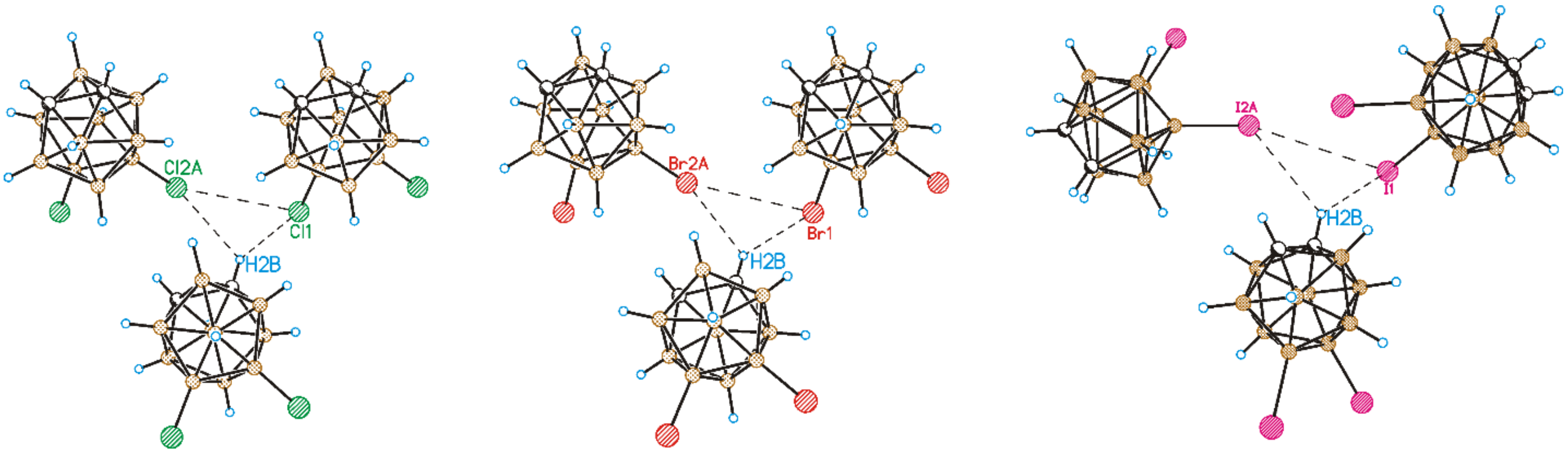

Figure S10. View of trimeric associates in 9,12-dichloro-, 9,12-dibromo- and 9,12-diiodo- (from left to right) derivatives of *ortho*-carborane. Chloro- and bromo-derivatives are isostructural while crystal packing of iodo-derivative is different. Trimeric associate of iodo-derivative can be considered only from the formal point of view: only I1…H2B contact is shorter than sum of van-der-Waals radii while I1…I2A and I2A…H2B contacts are significantly longer.
